# Review of clinical and diagnostic imaging of the thymus: from age-related changes to thymic tumors and everything in between

**DOI:** 10.1007/s11604-023-01497-w

**Published:** 2023-10-06

**Authors:** Daisuke Yamada, Masaki Matsusako, Yasuyuki Kurihara

**Affiliations:** Department of Radiology, St. Luke’s International Hospital, 9-1 Akashi-Cho, Chuo-Ku, Tokyo, 104-8560 Japan

**Keywords:** Thymus, Mediastinum, Prevascular space, International Thymic Malignancy Interest Group

## Abstract

The thymus, a primary lymphoid organ of the immune system, undergoes several changes due to a variety of reasons, ranging from aging to pathological conditions. These changes can make distinguishing between benign and neoplastic changes in the thymus challenging, thereby complicating the histopathological diagnoses of thymic tumors. Moreover, most patients with thymic tumors are asymptomatic at the time of diagnosis. Therefore, imaging plays an extremely important role in the evaluation of thymic lesions. In this review, we introduced the imaging characteristics of the thymus, ranging from benign findings, such as normal maturation and benign lesions, to neoplasms.

## Introduction

The mediastinum is the area between the left and right lungs, which contains the heart, great vessels, trachea, esophagus, thymus, and other organs [1]. Mediastinal tumors are generally defined as tumors that occur in the mediastinal organs. Mediastinal tumors are relatively rare, may be malignant or benign, and can occur in individuals across a wide range of age groups. Patients with mediastinal tumors are often asymptomatic when the tumors are small. In fact, approximately half of all mediastinal tumors are incidentally detected on chest X-rays or computed tomography (CT) scans. In addition, approximately 80% of asymptomatic mediastinal tumors are benign [2].

Usually, to diagnosis mediastinal tumors initial imaging studies are performed using a combination of chest radiography, chest CT, chest magnetic resonance imaging (MRI), and ultrasonography [3]. Some types of mediastinal tumors show characteristic abnormalities in blood tests, which are useful markers for diagnosis.

According to a previous report, the most common mediastinal tumors are thymomas, which account for approximately 40% of all mediastinal tumors [4].

In this article, we reviewed the imaging features of thymic tumors and the most frequently encountered mediastinal tumors, ranging from benign or malignant lesions that occurred during the normal maturation of the thymus to other anterior mediastinal tumors.

### Function, origin, and location of the thymus

The thymus is the main organ of the lymphatic system [5] and is relatively large from infancy to adolescence. However, it begins to decrease in size after puberty and continues to decrease further with increasing age. The primary function of the thymus, which is located in the upper chest, is to promote the development of T lymphocytes. T lymphocytes are white blood cells that protect against foreign organisms (bacteria and viruses) that infect body cells [6]. T lymphocytes also protect the body by controlling cancerous cells.

Hematopoietic stem cells are found in the bone marrow after birth. Granulocytes and B cells, such as red blood cells and platelets, are produced in organs where hematopoietic stem cells are found. In contrast, only T cells are produced in the thymus both in the fetus and after birth [7].

The thymus has a dual embryonic origin [8]. During the sixth week of gestation, the thymic epithelium develops from the ventral diverticular epithelium of the third pharyngeal pouch, along with the thyroid and parathyroid glands. It extends posterolaterally into the surrounding mesoderm as two flask-like structures. The cells that line these flask-like structures proliferate further and are eventually surrounded and invaded by the mesoderm. During the eighth week of gestation, the thymus descends and assumes its final position in the anterosuperior mediastinum. Thereafter, it fuses with its counterpart on the opposite side. Hematopoietic bone marrow precursor cells (mesenchymal origin) migrate into the thymus later in the developmental process. This is how thymocytes reach the thymus gland, and the lymphoid tissue merges with the epithelial cell framework of the thymus [9]. The growth and development of the thymus continue until puberty.

### Thymus location

**Fig. 1:**
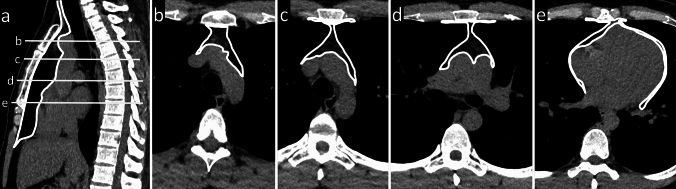
International Thymic Malignancy Interest Group definition of the prevascular compartment. The prevascular compartment is the area marked by the white line

The mediastinum contains vital vascular and nonvascular structures and organs. The mediastinum is divided into specific compartments that are valuable for the identification, characterization, and management of various mediastinal abnormalities. Numerous mediastinal compartment classification systems have been developed and are used to varying degrees by anatomists, surgeons, and radiologists. In 2014, the Japanese Association for Research on the Thymus (JART) developed a four-compartment multidetector CT–based classification scheme for the division of the mediastinal compartments [1]. Based on discussions with experts in the field of mediastinal diseases, the International Thymic Malignancy Interest Group (ITMIG) modified the JART model, introduced a new definition of mediastinal compartments for cross-sectional imaging, and adopted it as a new standard [10]. The thymus is located in a prevascular compartment according to the ITMIG classification (Fig. 1). The following boundaries of the prevascular compartment are defined in the ITMIG classification system: (1) superiorly, the thoracic inlet; (2) inferiorly, the diaphragm; (3) anteriorly, the posterior border/cortex of the sternum; (4) laterally, the parietal mediastinal pleura; and (5) posteriorly, the anterior aspect of the pericardium that wraps around the heart in a curvilinear fashion. Based on these landmarks, the major components of the prevascular compartment include the thymus, fat, lymph nodes, and the left brachiocephalic vein. The most common abnormalities encountered in the prevascular compartment include thymic lesions (cysts, hyperplasia, and malignancies, such as thymomas, thymic carcinomas, and neuroendocrine neoplasms), germ cell neoplasms (which arise from germ cell remnants in the mediastinum), lymphomas, metastatic lymphadenopathy, and intrathoracic goiters.

### Age-specific changes in the thymus

**Fig. 2:**
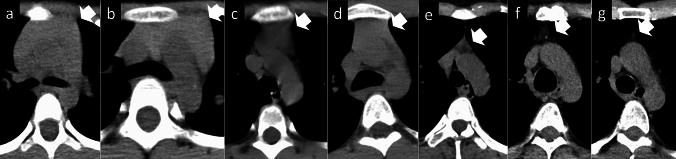
Variations in the thymus size with age. In infants, the thymus has a square-to-trapezoid mass-like form. As age increases, the thymus changes to an arrowhead shape, atrophies, and almost completely regresses by the age of 50. The thymus in a **a** 2-year-old, **b** 5-year-old, **c** 10-year-old, **d** 15-year-old, **e** 20-year-old, **f** 35-year-old, and **g** 50-year-old. Arrows indicate the thymus

The size of the thymus varies greatly with age. In infants, the thymus has a square to trapezoid mass-like form [11]. As age increases, it changes to an arrowhead shape, atrophies, and almost completely regresses by the age of 50 [12] (Fig. 2).

### Thymic hyperplasia

Thymic hyperplasia can be subdivided into two types: true thymic hyperplasia and lymphoid thymic hyperplasia [13]. True thymic hyperplasia refers to an enlarged thymus gland with an increase in normally organized thymus tissue, exceeding the upper limit of normal for a specific age, as determined by weight and volume. This condition occurs in patients recovering from recent stressors such as anticancer chemotherapy, corticosteroid therapy, radiotherapy, or thermal burns. After being initially atrophic, the thymus gland regrows once the stress is relieved. In some cases, the thymus continues to grow and becomes larger than its original size, known as "rebound hyperplasia". On the other hand, lymphoid hyperplasia is characterized by an increased number of lymphoid follicles and germinal centers in the thymus. The size of the thymus gland may not always be enlarged and can even be atrophic or involved in a neoplasm. Lymphoid hyperplasia is commonly associated with various immunologically mediated diseases, particularly myasthenia gravis.

Both true and lymphoid thymic hyperplasia manifest as a diffuse symmetric enlargement of the thymus, making it difficult to distinguish between them based on imaging findings alone [13]. It is important to distinguish thymic hyperplasia from neoplasms, which tend to present as focal masses. Inaoka et al. [14] reported that thymic hyperplasia could be differentiated from neoplastic lesions using dual-echo chemical shift imaging. A chemical shift ratio (CSR) is calculated by comparing the signal intensity of the thymus gland (tSI) with that of the paraspinal muscle (mSI) on both in-phase (in) and opposed-phase (op) images using the following formula:

$${\text{CSR}}\, = \,\left( {{\text{tSIop}}/{\text{mSIop}}} \right)/\left( {{\text{tSIin}}/{\text{mSIin}}} \right).$$

**Fig. 3:**
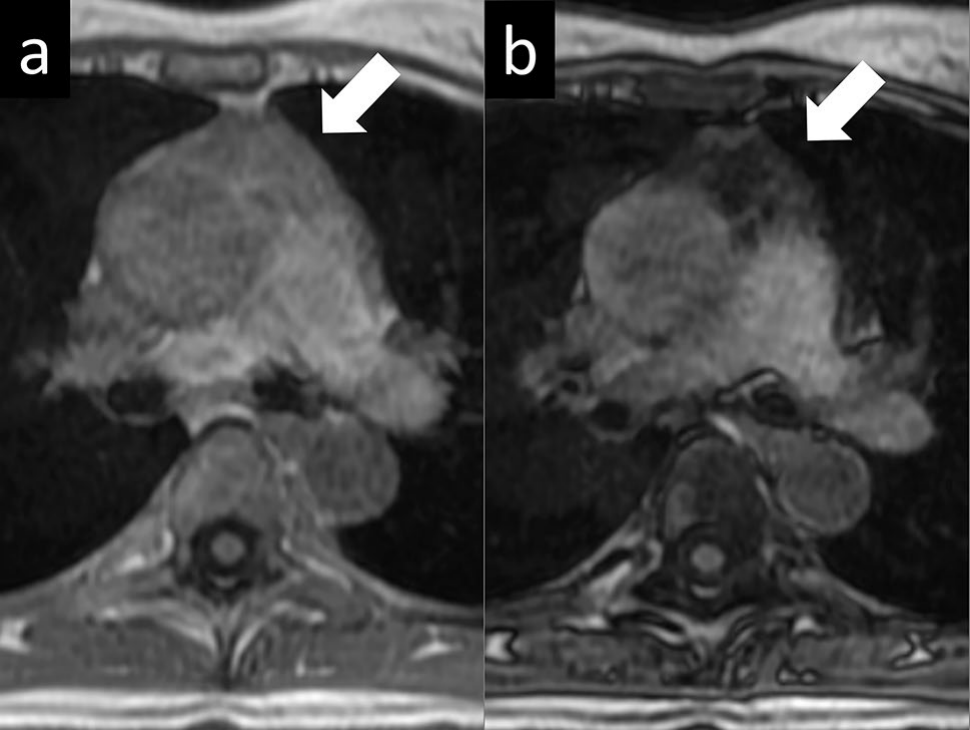
Thymic hyperplasia. A 40-year-old woman with thymic hyperplasia. Transverse in-phase **a** and opposed-phase **b** gradient-echo T1-weighted MR images indicate an apparent decrease in the signal intensity of the thymus on the opposed-phase image relative to the in-phase image (arrows). The CSR is 0.45. MR, magnetic resonance; CSR, chemical shift ratio

The mean CSR was 0.61 in the hyperplasia group and 1.0 in the tumor group (Fig. 3) [15]. Another useful quantitative parameter is the signal intensity index (SII), which is calculated as follows:

$${\text{SII}}\, = \,\left[ {\left( {{\text{tSIin}}\, - \,{\text{tSIop}}} \right)/\left( {{\text{tSIin}}} \right)} \right]\, \times \,{1}00\% .$$

Priola et al. [16] reported that SII is more practical than CSR for evaluating thymic abnormalities using single-breath-hold acquisition dual-echo imaging. They reported that SII (cutoff: 8.92%) had 100% sensitivity and 100% specificity in differentiating thymic tumors from thymic hyperplasia [16]. Given that dual-echo acquisition is resistant to misregistration and does not require normalization with a reference tissue, the use of SII is recommended instead of CSR because it is easier to calculate and is obtained using a more robust technique.

The CSR is influenced by patient age and weight and tends to be lower in younger and underweight individuals than in older adults and overweight patients because individuals in the latter groups have a higher intramuscular lipid content. Chemical-shift MRI exploits the differences in resonance between fat and water protons. One caveat is that the opposed-phase images of thymolipomas may show signal suppression. Conversely, the opposed-phase images of a normal or hyperplastic thymus in children, young adults, and lean patients may not show signal suppression because of the low amount of intercalated fat in the gland. Thus, caution should be exercised during the evaluation of a non-suppressed opposed-phase image of the thymus in patients who are likely to have a benign-appearing lipid-poor thymus.

### Thymic cysts

**Fig. 4:**
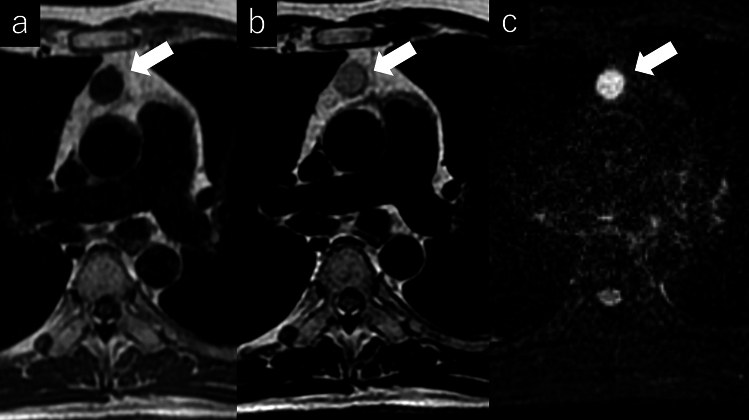
Thymic cyst. A 51-year-old man with thymic cyst. A 2-cm round cyst is observed in the anterior mediastinum. **a** T1WI shows low signal intensity (arrow). **b** T2WI shows a slightly high signal intensity (arrow). **c** Fat-suppressed T2WI shows a markedly high signal intensity (arrow). The cyst is a well-defined, unifocal lesion with serous content. T1WI, T1-weighted image; T2WI, T2-weighted image

The thymus migrates caudally during development and is ultimately located in the anterior mediastinum [17]. Thymic cysts arise from the tissue left over during this process and can occur anywhere from the lower neck to the upper mediastinum. Most thymic cysts are congenital, although some are acquired. Acquired thymic cysts may occur after radiation therapy for malignant lymphoma, open-heart surgery, Sjögren’s syndrome, lymphoproliferative disorders, or human immunodeficiency infection. The multifocal form of acquired thymic cysts is often secondary to inflammatory changes [18]. Acquired thymic cysts may present as multilocular thymic cysts, which can be problematic to differentiate from mucosa-associated lymphoid tissue (MALT) lymphoma. The cysts are more common in males and most patients are asymptomatic (Fig. 4). In addition, most of the cysts have serous contents. Congenital cysts contain a mildly hemorrhagic fluid and have a slightly high CT value. In addition, most of the cysts have thin walls. Differential diagnoses for thymic cysts include pericardial cysts, thymomas with strong cystic degeneration, and mature cystic teratomas.

### Thymomas

Thymic tumors are rare; however, since they are the most common anterior mediastinal tumors, it is important to understand their clinical and imaging presentations. Specifically, the clinical features of thymomas need to be fully understood because they are the most common type of thymic tumors [19].

The clinical manifestations of thymomas are almost asymptomatic, although sometimes there are subjective symptoms (cough, chest pain, dyspnea, dysphagia, hoarseness, and superior vena cava syndrome) due to extrinsic compression. In addition, conditions such as myasthenia gravis (or a certain percentage of patients with myasthenia gravis have thymic tumors), pure red cell aplasia, hypogammaglobulinemia, collagen disorders and inflammatory bowel disease may occasionally accompany the thymoma [19].

**Fig. 5:**
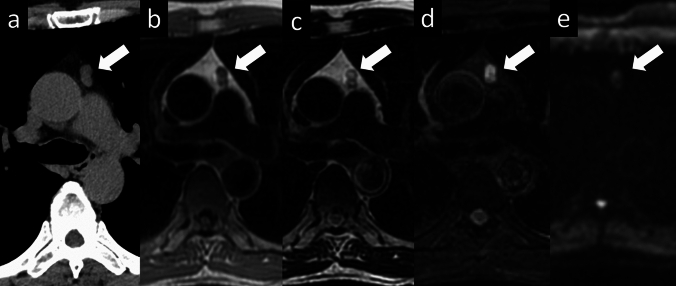
Thymoma (type A). A 73-year-old man with type A thymoma. **a** Chest CT scan shows a 1.3-cm, slightly lobulated, prevascular nodule (arrow). **b**, **c** MRI shows a prevascular nodule with low signal intensity on T1WI **b** and high signal intensity on T2WI **c** (arrow). **d** MRI shows high intensity on fat-suppressed T2WI (arrow). **e** MRI shows slightly high signal intensity on DWI (arrow) and a high ADC value (3.4 × 10^–3^ mm^2^/s). The pathological diagnosis was type A thymoma. CT, computed tomography; MRI, magnetic resonance imaging; T1WI, T1-weighted image; T2WI, T2-weighted image; DWI, diffusion-weighted imaging; ADC, apparent diffusion coefficient

**Fig. 6:**
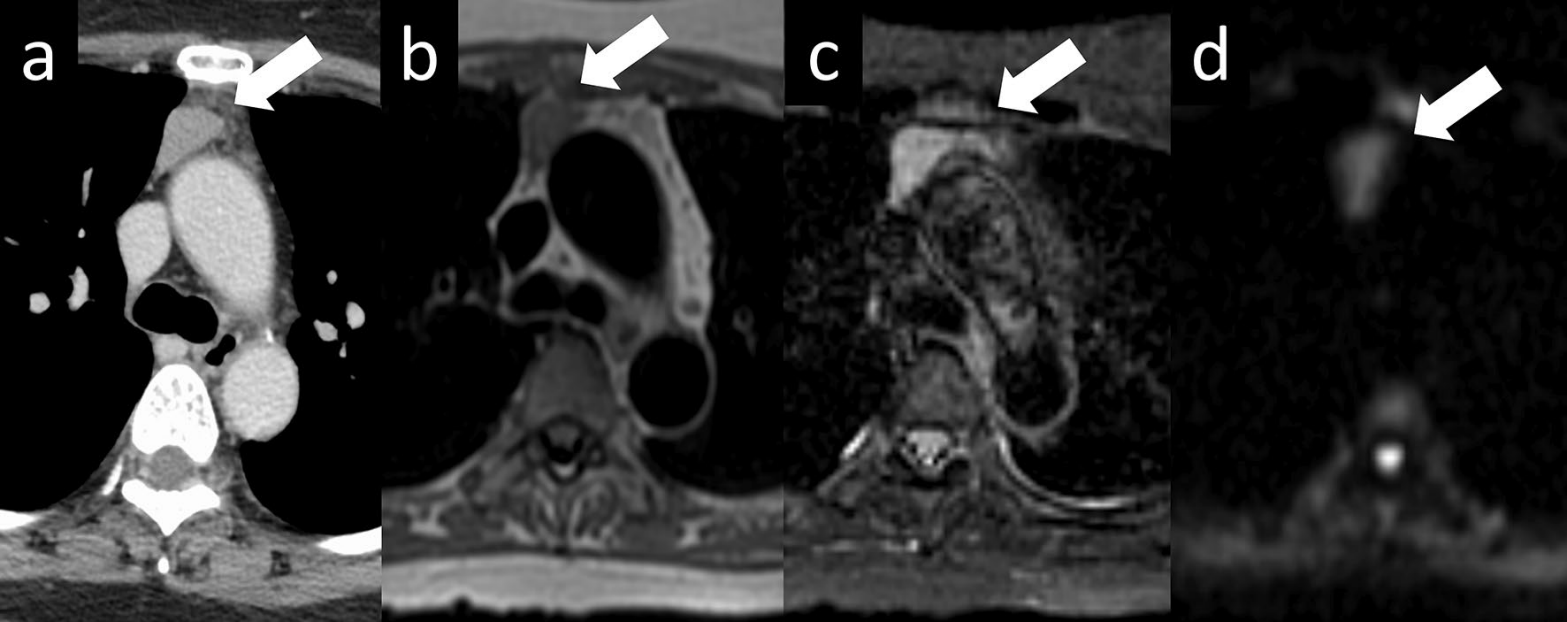
Thymoma (type AB). A 50-year-old woman with type AB thymoma. **a** Chest CT scan shows a 2-cm prevascular nodule with well-defined borders (arrow). **b**–**d** MRI shows a slightly indistinct border with perivascular fat on the left side of the nodule and a slightly low ADC value (1.36 × 10^–3^ mm^2^/s) (arrow) (**b**: T1WI, **c**: fat-suppressed T2WI, **d**: DWI). CT, computed tomography; MRI, magnetic resonance imaging; ADC, apparent diffusion coefficient, T1WI, T1-weighted image; T2WI, T2-weighted image; DWI, diffusion-weighted imaging

**Fig. 7:**
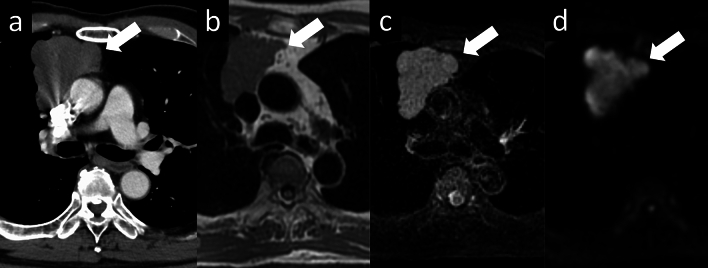
Thymoma (type B1). A 73-year-old man with type B1 thymoma. **a** Chest CT scan shows a 7-cm, lobulated, prevascular mass (arrow). **b**–**d** MRI shows high intensity on fat-suppressed T2WI and a slightly low ADC value (1.37 × 10^–3^ mm^2^/s) (arrow) (**b**: T1WI, **c**: fat-suppressed T2WI, **d**: DWI). CT, computed tomography; MRI, magnetic resonance imaging; T2WI, T2-weighted imaging; ADC, apparent diffusion coefficient; T1WI, T1-weighted imaging; DWI, diffusion-weighted imaging

**Fig. 8:**
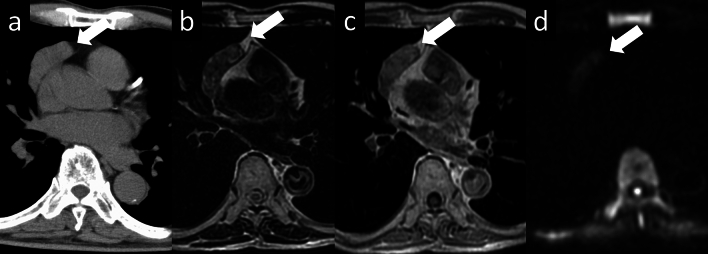
Thymoma (type B2). An 80-year-old man with type B2 thymoma. **a** Chest CT scan shows a 5-cm, slightly lobulated, prevascular mass (arrow). **b**–**d** MRI shows heterogeneous signal intensity on T2WI, heterogeneous enhancement on contrast-enhanced T1WI, and a low ADC value (1.13 × 10^–3^ mm^2^/s) (arrow) (**b**: T2WI, **c**: contrast-enhanced T1WI, **d**: DWI). CT, computed tomography; MRI, magnetic resonance imaging; T2WI, T2-weighted imaging; T1WI, T1-weighted imaging; ADC, apparent diffusion coefficient; DWI, diffusion-weighted imaging

**Fig. 9:**
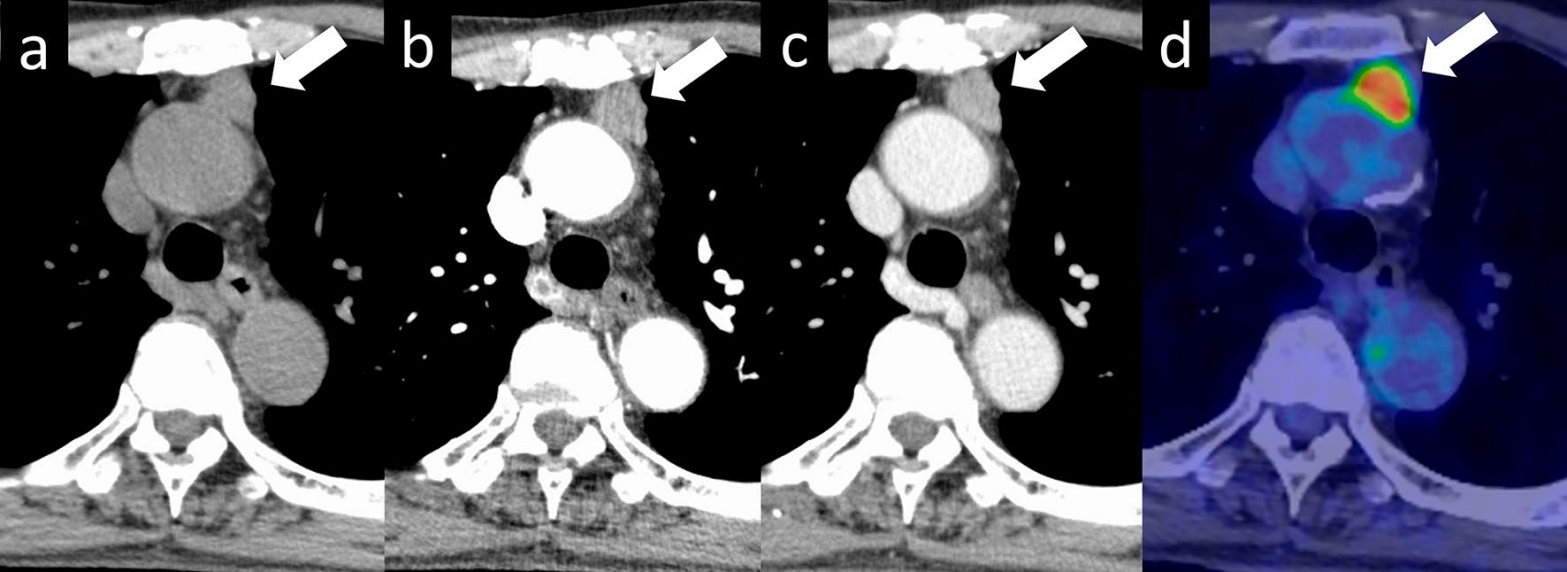
Thymoma (type B3). A 71-year-old man with type B3 thymoma. **a**–**c** On contrast-enhanced dynamic CT, a 2-cm, lobulated nodule with homogenous attenuation shows gradual enhancement (arrow). The internal density of the nodule is homogeneous. Contrast-enhanced dynamic CT of the nodule shows gradual enhancement. **d** 18F-FDG PET shows high FDG uptake in the perivascular nodule (SUVmax = 9.74) (arrow). CT, computed tomography; 18F-FDG PET, 18F-fluorodeoxyglucose positron emission tomography; SUVmax, maximum standardized uptake value

The histopathological subtypes of thymic epithelial tumors are diverse. The World Health Organization (WHO) classification of the histopathology of thymic tumors is widely used. The clinical grades and prognoses of the histological subtypes of thymic tumors differ [20, 21]. Two major histological types of thymomas are recognized: types A and B. For type A thymoma, the neoplastic epithelial cells and their nuclei are conical or oval, homogeneous, and mildly atypical. Type B thymomas are further classified into three types according to the degree of lymphocytic involvement and atypia of the neoplastic epithelial cells (Table 1). In the fifth edition of the WHO classification, which was published in 2021, the classification of thymomas and thymic carcinomas was strongly reinforced based on new molecular findings [21]. The Cancer Genome Atlas study revealed that types A and AB thymomas and types B1 to B3 thymomas each belong to a spectrum of tumors, with minimal overlap between them (Figs. 5, 6, 7, 8 and 9). These groups are genomically distinct from thymic carcinomas. The Masaoka-Koga staging system [22], which determines the clinical stage of thymomas according to the degree of local invasion, is correlated with prognosis and is widely used (Table 2). The editors of the 5th edition prompt the adoption of the TNM system as the mandatory staging system for thymic epithelial tumor and the modified Masaoka-Koga system as optional [20, 21].

**Table 1:** World Health Organization classifications of thymomas

	Type A	Type AB	Type B1	Type B2	Type B3
Epithelial cells	Spindle	Mixed (type A and B)	Spindle + polygonal	Polygonal	Polygonal
Lymphocytes	Less	A few more	More	A few more	Less
Prognosis	Good Poor

**Table 2:** Masaoka-Koga and TNM classifications

TNM classification			Masaoka-Koga stage	Criteria
T	N	M		
1a	0	0	I	Grossly and microscopically completely encapsulated tumor
1a	0	0	IIa	Microscopic transcapsular invasion
1a	0	0	IIb	Macroscopic invasion into surrounding fatty tissue
1b	0	0	III	Macroscopic invasion into neighboring organ T1b: mediastinal pleura T2: pericardium T3: lung, brachiocephalic vein, superior vena cava, phrenic nerve, chest wall, extra pericardial pulmonary vein T4: aorta, arch vessels, intrapericardial pulmonary artery, myocardium, trachea, oesophagus
2	0	0		
3	0	0		
4	0	0		
1–4	0	1a	IVa	Pleural or pericardial metastases
1–4	1	0-1b	IVb	Lymphogeneous or hematogeneous metastasis N1: metastasis in anterior (perithymic) lymph nodes N2: metastasis in deep intrathoracic or cervical lymph nodes M1a: separate pleural or pericardial nodule(s) M1b: pulmonary intraparenchymal nodule or distant organ metastasis
1–4	2	0-1b		
1–4	0–2	1b		

TNM, tumor, nodes, and metastasis

The three basic imaging modalities used for the evaluation of thymomas are CT, MRI, and positron emission tomography (PET). Certain key points should be considered in the diagnosis of thymoma using each imaging modality. For CT, localization, calcification, marginal features, and surrounding invasion should be studied. For MRI, the internal signal, especially the diffusion-weighted imaging/apparent diffusion coefficient (ADC) map, should be closely evaluated [23]. Regarding PET, the maximum standardized uptake value (SUVmax) should be assessed [24]. Types A and AB thymomas typically show a smooth contour on CT and MRI scans, with a distinct low-signal-intensity capsule surrounding the mass on T2-weighted images [4]. Type B1, B2 and B3 thymomas often exhibit an irregular contour, with less defined capsules and septa compared to type A and AB thymomas. Types B1, B2, and B3 thymomas frequently show calcifications, particularly linear or curvilinear calcifications along septa. Pleural dissemination is common in high-risk thymomas. 18F- Fluorodeoxyglucose (FDG) accumulation of type A and AB thymomas are low. Type B1, B2 and B3 thymomas exhibit various degrees of accumulation and a definite conclusion has not yet been reached; however, overall, the higher the histological grade, the higher the 18F-FDG accumulation becomes, suggesting that this is correlated to staging [24].

**Fig. 10:**
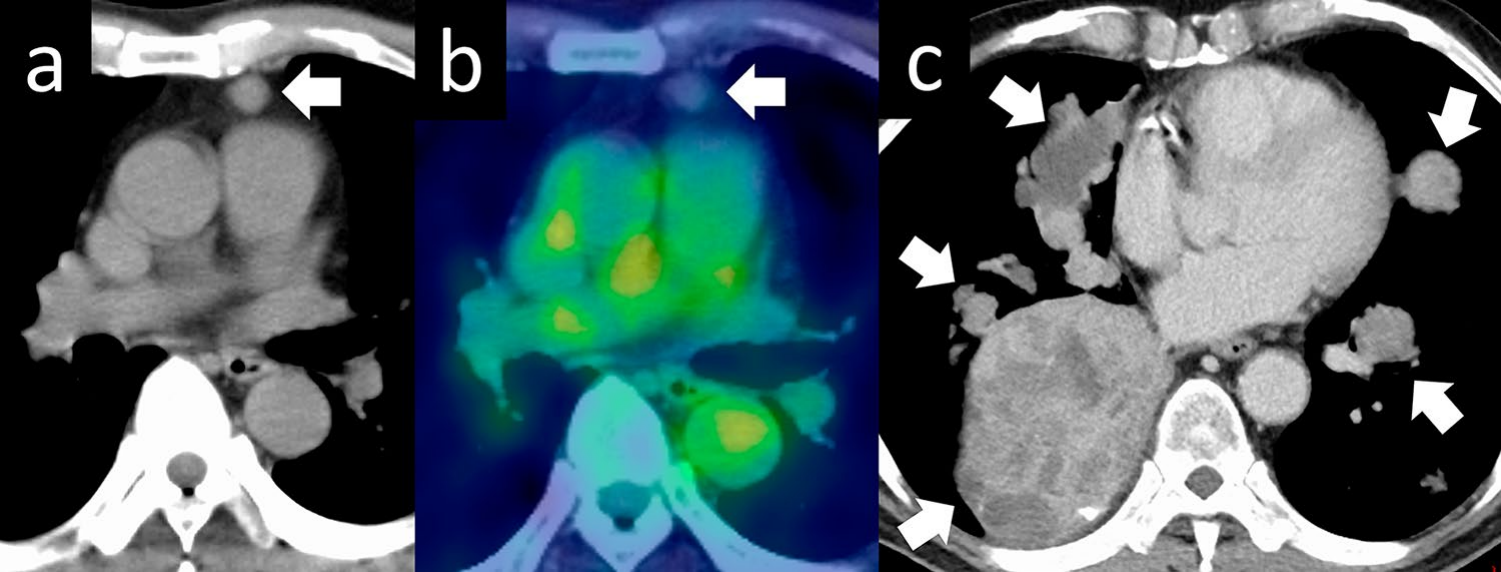
Atypical type A thymoma. A 64-year-old man with an atypical subtype of type A thymoma. **a** Chest CT shows a small round nodule in the anterior mediastinum that did not invade the surrounding organs (arrow). **b** 18F-FDG PET shows no hypermetabolism in the nodule (arrow). **c** CT scan is taken 10 years after surgery. The patient relapses and has multiple lung, bone, and brain metastases (arrows). CT, computed tomography; 18F-FDG PET, 18F-fluorodeoxyglucose positron emission tomography

The thymoma grade can be estimated to some extent using imaging findings. However, it should be noted that in rare cases, thymomas with imaging findings similar to those of low-grade thymomas may have poor prognoses (Fig. 10). An atypical type A thymoma variant was added to the WHO classification of the type A thymoma family in 2015 based on several reports of type A thymomas that showed oncologically aggressive behavior and tumor relapses. In the 2021 WHO revision, the atypical variant was changed to an atypical subtype. Its imaging findings indicate that it is a low-grade thymoma that show no evidence of invasion into surrounding tissues on CT and a low SUVmax on 18 F-FDG PET. In contrast, its pathological findings are specific features of atypical type A thymomas. The findings are as follows: (1) mild-to-moderate nuclear atypia, (2) increased mitotic activity, and (3) scattered foci of necrosis. Some cases require follow-up even when imaging findings suggest low-grade thymoma. Thus, careful discussion with a pathologist is necessary.

**Fig. 11:**
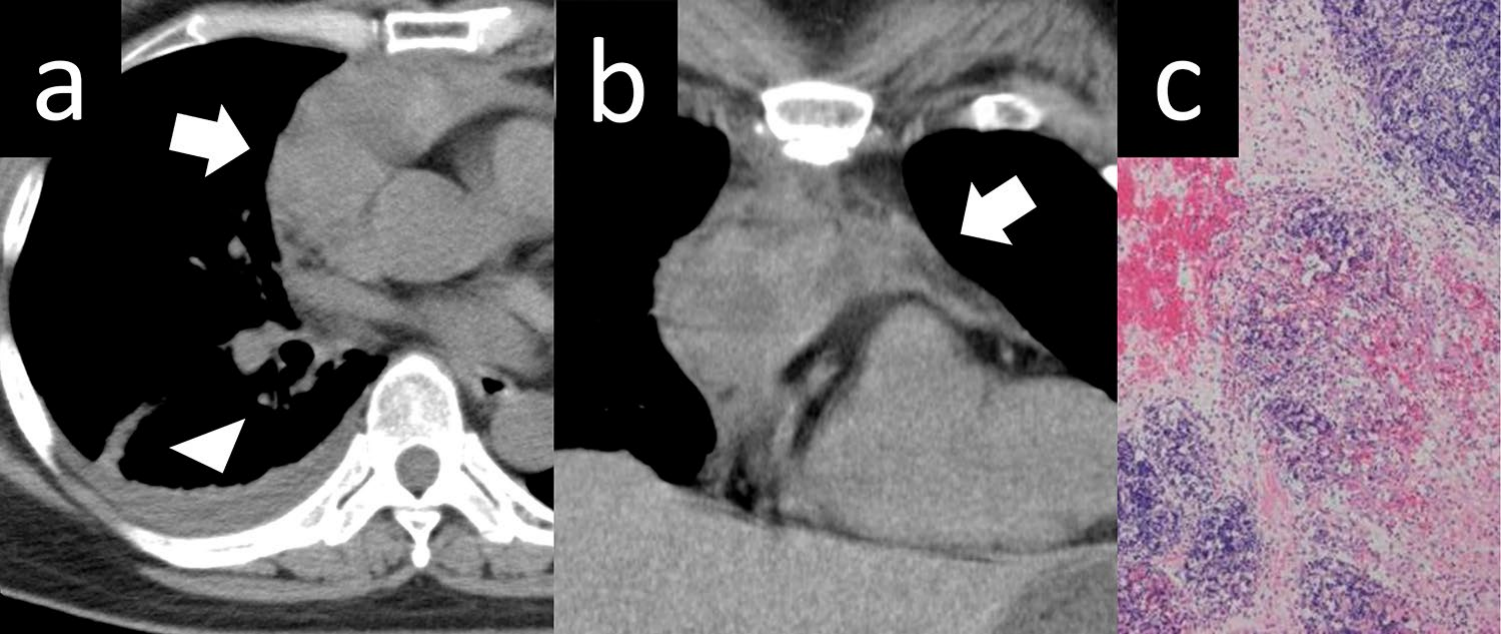
Ruptured thymoma. A 50-year-old woman with ruptured thymoma. **a**, **b** Chest CT shows a 6-cm, lobulated, perivascular mass (arrow). An uneven increase in the density of fatty tissue in the mediastinum is noted, with a slightly higher pleural effusion density (arrowhead). **c** The pathological diagnosis is type B2 thymoma. The tumor shows internal necrosis and inflammation that spread to the surrounding tissues (HE staining, medium-power image). CT, computed tomography; HE, hematoxylin and eosin

**Fig. 12:**
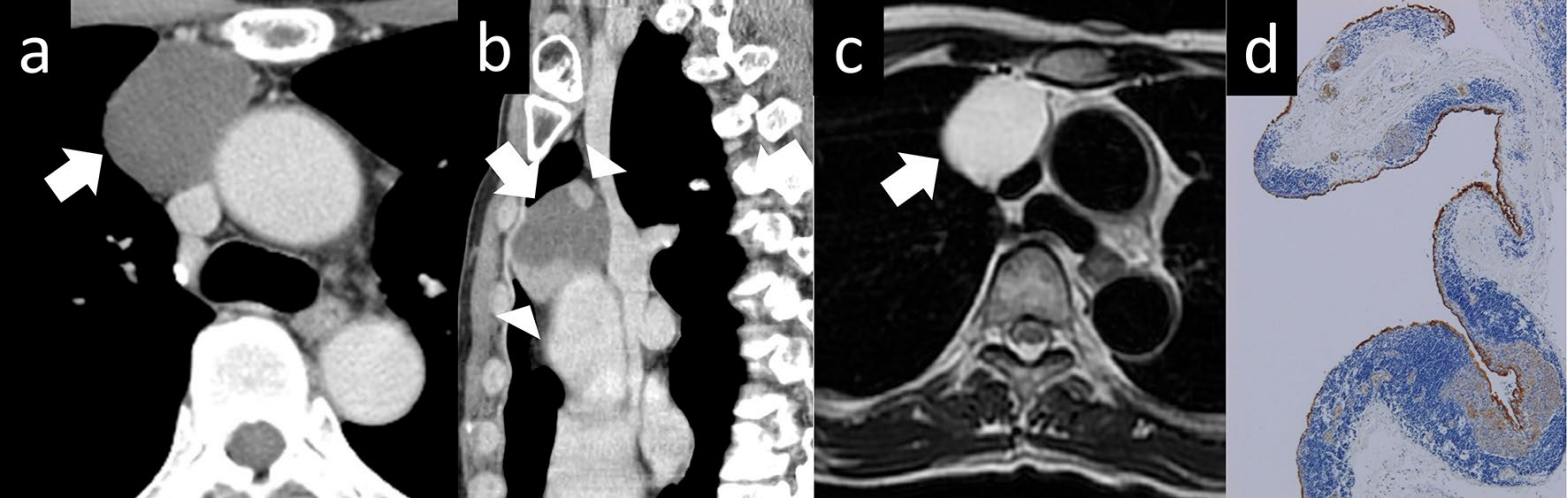
Cystic thymoma. A 60-year-old man with cystic thymoma. **a**, **b** Contrast-enhanced CT shows a 4-cm cystic mass (arrow). Enhanced nodules are observed within the cyst (arrowheads). **c** The cystic mass shows high intensity on T2WI (arrow). **d** The pathological diagnosis was type B2 cystic thymoma. The brown staining represents the epithelial cells, which constitute the cyst wall. Conversely, the blue-stained area corresponds to abnormal cells, which are characteristic of a thymoma. The cyst wall is covered with an epithelial layer. The mass is considered a thymoma arising from a congenital thymic cyst (Cytokeratin staining, low-power image). CT, computed tomography; T2WI, T2-weighted imaging

Additionally, thymomas with nonspecific imaging findings, such as rupture or cystic changes, may also be encountered (Figs. 11 and 12) [25, 26]. Thymomas are rarely associated with hemorrhage or rupture. Hemorrhage and necrosis are assumed to occur in the tumors because of hematological disturbances caused by chronic inflammation. Ruptures of mediastinal thymomas are a recognized cause of chest pain and massive pleural effusion. Thymic cysts can be classified as congenital or acquired. Congenital thymic cysts are believed to be remnants of the thymopharyngeal duct. In addition, epithelium is observed on the cyst wall. Acquired thymic cysts are usually multifocal and are associated with inflammatory findings. Cystic changes in thymomas are differentiated by the lack of epithelium on the cyst wall. Although thymic cysts are often reported, thymomas and thymic carcinomas arising from cyst walls have been reported as well; therefore, caution should be exercised during diagnosis.

### Thymic carcinomas

**Fig. 13:**
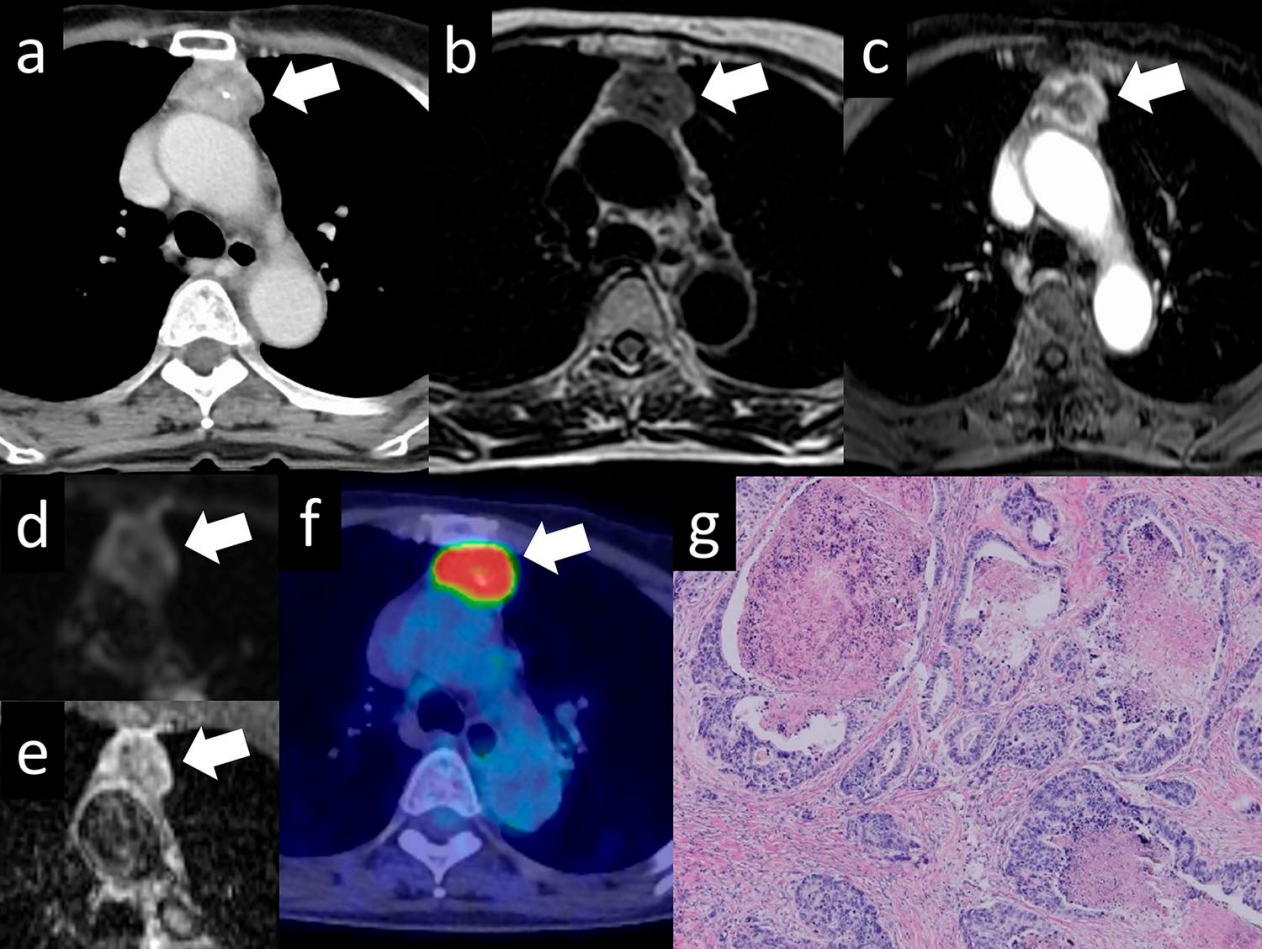
Thymic squamous cell carcinoma. A 60-year-old woman with thymic squamous cell carcinoma. **a** On contrast enhanced chest CT, a 4.4-cm mass with small calcification shows heterogeneous enhancement (arrow) **b** The mass shows low intensity on T2WI (arrow). **c** The mass shows heterogeneous intensity on contrast-enhanced T1WI. **d**, **e** The mass shows a low ADC value (1.1 × 10^–3^ mm^2^/s) (arrow). **f** 18F-FDG PET shows high FDG uptake in the perivascular mass (SUVmax = 10.1) (arrow). **g** The pathological diagnosis is thymic squamous cell carcinoma (HE staining, medium-power image). The low signal intensity area in the thymic mass on T2WI indicates collagenous tissue and fibrosis pathologically. CT, computed tomography; T2WI, T2-weighted imaging; T1WI, T1-weighted imaging; ADC, apparent diffusion coefficient; 18F-FDG PET, 18F-fluorodeoxyglucose positron emission tomography; SUVmax, maximum standardized uptake value; HE, hematoxylin and eosin

**Fig. 14:**
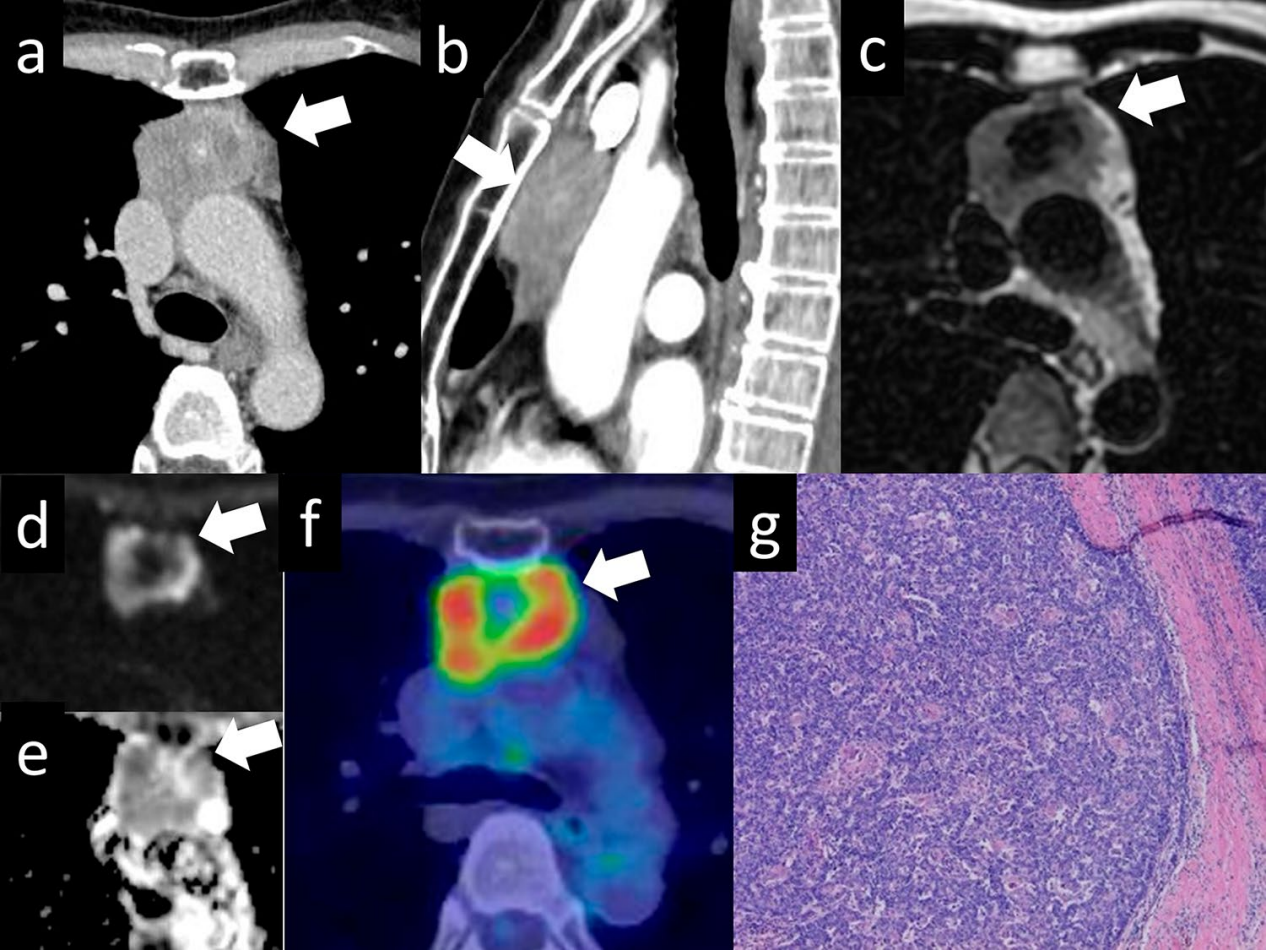
Thymic adenocarcinoma. A 70-year-old woman with a thymic adenocarcinoma. **a**, **b** On contrast enhanced chest CT, a 3-cm mass with small calcification shows heterogenous enhancement (arrow). **c** The mass shows low intensity on T2WI (arrow). **d**, **e** The mass shows a low ADC value (1.2 × 10^–3^ mm^2^/s) (arrow). **f** 18F-FDG PET shows high FDG uptake in the perivascular mass (SUVmax = 12.73) (arrow). **g** The pathological diagnosis is thymic adenocarcinoma (HE staining, medium-power image). CT, computed tomography; T2WI, T2-weighted imaging; ADC, apparent diffusion coefficient; 18F-FDG PET, 18F-fluorodeoxyglucose positron emission tomography; SUVmax, maximum standardized uptake value; HE, hematoxylin and eosin

Thymic carcinomas are more aggressive than thymomas and have a higher tendency to spread to other organs. They often exhibit more cellular atypia, increased mitotic activity, and a higher propensity for invasion into nearby structures. The most common histological type of thymic carcinoma is squamous cell carcinoma (Fig. 13). Thymic carcinomas can also present as adenocarcinomas (Fig. 14); however, it is important to distinguish them from metastatic tumors. Thymic carcinomas have a higher tendency to invade surrounding organs, such as mediastinal blood vessels, pleura, lungs, and pericardium, and have a poorer prognosis than thymomas [27]. A low-signal-intensity area on T2-weighted images of a thymic mass is considered suggestive of thymic carcinoma. Histopathologically, these findings may reflect the presence of collagenous tissue or fibrosis. The three characteristic imaging findings of thymic epithelial tumors that are indicative of possible malignancy are a low-signal area on T2WI, low value on the ADC map, and increased 18F-FDG uptake [28].

### Thymic neuroendocrine neoplasms

Thymic neuroendocrine neoplasms (TNENs) are rare tumors that originate in the thymus [29]. These tumors are a subset of neuroendocrine tumors (NETs), which arise from neuroendocrine cells that produce hormones and are scattered throughout the body. TNENs are classified into two categories: NETs (typical carcinoids [NETs, grade 1] or atypical carcinoids [NETs, grade 2]) and neuroendocrine carcinomas (NECs) (small cell carcinomas or large cell NECs). Typical carcinoids are low grade-malignancies, while atypical carcinoids and NECs are considered high-grade malignancies. The behavior and prognosis of these tumors depend on their histological characteristics.

**Fig. 15:**
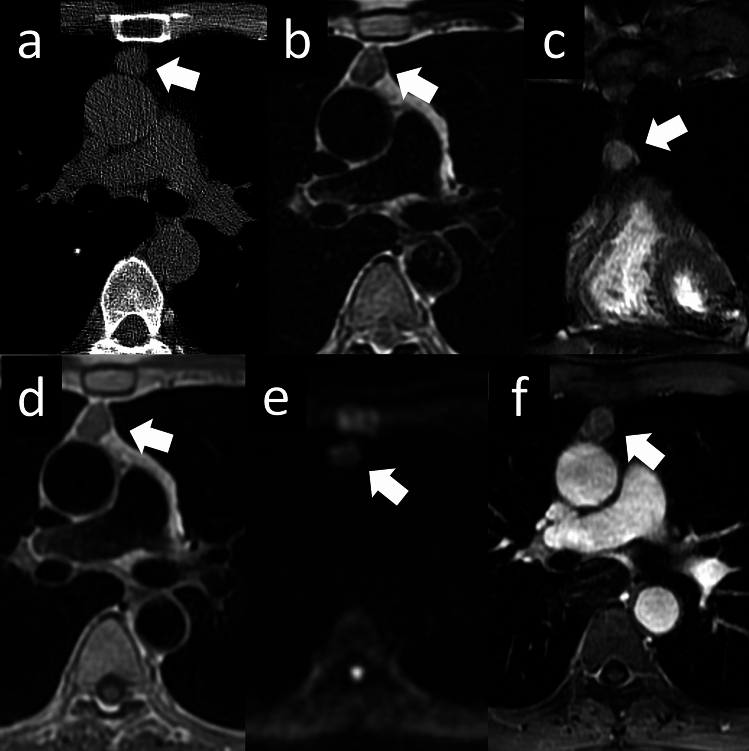
Thymic neuroendocrine neoplasm. A 50-year-old woman with a thymic neuroendocrine neoplasm. **a** Chest CT scan shows a 2.0-cm nodule (arrow). **b**–**d** MRI shows heterogeneous signal intensity on T2WI (**b**: axial, **c**: coronal) and T1WI (**d**). **e** MRI shows slightly high signal intensity on DWI (arrow). **f** MRI shows a slight enhancement on contrast-enhanced T1WI (arrow). CT, computed tomography; MRI, Magnetic Resonance Imaging; T2WI, T2-weighted imaging; T1WI, T1-weighted imaging; DWI, diffusion-weighted imaging

Imaging plays a crucial role in the diagnosis, staging, and management of TNENs [29]. Various imaging modalities can be utilized, including CT, MRI, and PET. CT scans are commonly used for the initial evaluation of TNENs. They provide detailed cross-sectional images of the thymus and surrounding structures. TNENs typically appear as well-defined, solid masses within the thymus (Fig. 15). These masses may show heterogeneous enhancement after the administration of contrast material. CT can also help assess the extent of tumor involvement, such as invasion into neighboring structures or the presence of distant metastases. MRI can provide additional information about TNENs, particularly regarding their local extent and invasion into adjacent structures. MRI is particularly useful in distinguishing between thymic tumors and other mediastinal masses, as it offers excellent soft tissue contrast. On MRI, TNENs usually appear as solid masses with variable signal intensity on T1- and T2-weighted images. Gadolinium-based contrast agents can be used to assess enhancement patterns, which may aid in differentiating TNENs from benign thymic lesions. PET scans, often combined with CT (PET/CT), are valuable tools for TNEN evaluation. These scans utilize a radioactive tracer, typically 18F-FDG, which accumulates in metabolically active tissues. TNENs with high metabolic activity will show increased uptake of the tracer. PET/CT can help detect small lesions, assess regional and distant metastases, and guide treatment planning. However, TNENs can exhibit variable 18F-FDG uptake, with some tumors showing low or no uptake, particularly in well-differentiated carcinoid tumors.

### Germ cell tumors (GCTs) of the mediastinum

Germ cell tumors (GCTs) account for approximately 20% of all mediastinal tumors and cysts. These tumors are believed to arise from extragonadal germ cells. Brown et al. [30] reported the development of extragonadal teratomas, in which embryonic cells that emerge during embryonic development migrate from behind the intestinal primordium to the germinal ridge, where they remain or stray, and develop into tumors. They are always associated with the thymus gland and may even be contained within it when small. Mediastinal GCTs are not related to thymomas [31]. The possibility of metastases from primary testicular or ovarian tumors must be excluded before a diagnosis of primary mediastinal GCT is made.

**Fig. 16:**
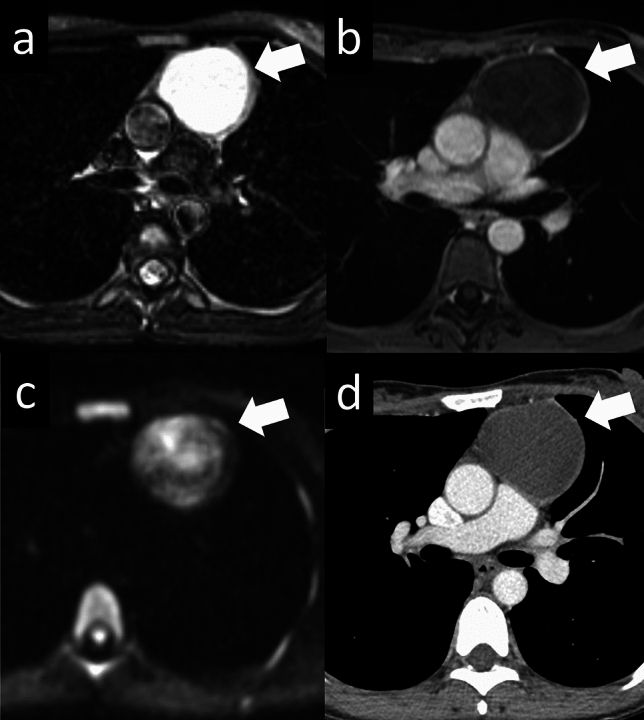
Mature cystic teratoma. A 35-year-old woman with mature cystic teratoma. **a** Fat-suppressed T2WI shows a mass lesion in the anterior mediastinum with high signal intensity (arrow). **b** Contrast-enhanced T1WI shows enhancement of the capsule (arrow). **c** DWI shows heterogeneous high signal intensity. **d** CT shows a 6.0 cm cystic mass (arrow). T2WI, T2-weighted imaging; T1WI, T1-weighted imaging; DWI, diffusion-weighted imaging; CT, computed tomography

Mediastinal GCTs are associated with Klinefelter syndrome [32]. There is an increased risk of thymic GCTs, particularly seminomas, in individuals with Klinefelter syndrome compared to the general male population. The exact mechanisms underlying this relationship are not fully understood, but it is believed that abnormal germ cell development in Klinefelter syndrome may contribute to tumor formation. However, it is important to note that not all individuals with Klinefelter syndrome develop thymic GCTs, and the majority of these tumors occur in individuals without Klinefelter syndrome. The histological subtype of mediastinal GCTs is related to the sex of the patient. Seminomas are observed only in males. Embryonal carcinoma, yolk sac tumor, teratocarcinoma, and choriocarcinoma show male predilection but may sometimes be observed in females. Mature cystic teratomas, the most common type of mediastinal germ cell neoplasms, occur with equal frequency in males and females [26]. Mature teratomas may contain a vast range of tissues and thus have variable imaging features. In general, mature teratomas are well-demarcated, have displacing rather than invading adjacent structures, are usually cystic, and show variable attenuation, fat and water densities, and calcification. Treatment depends on whether the teratoma is mature or immature. Surgical resection is curative in cases of mature teratomas (Fig. 16).

**Fig. 17:**
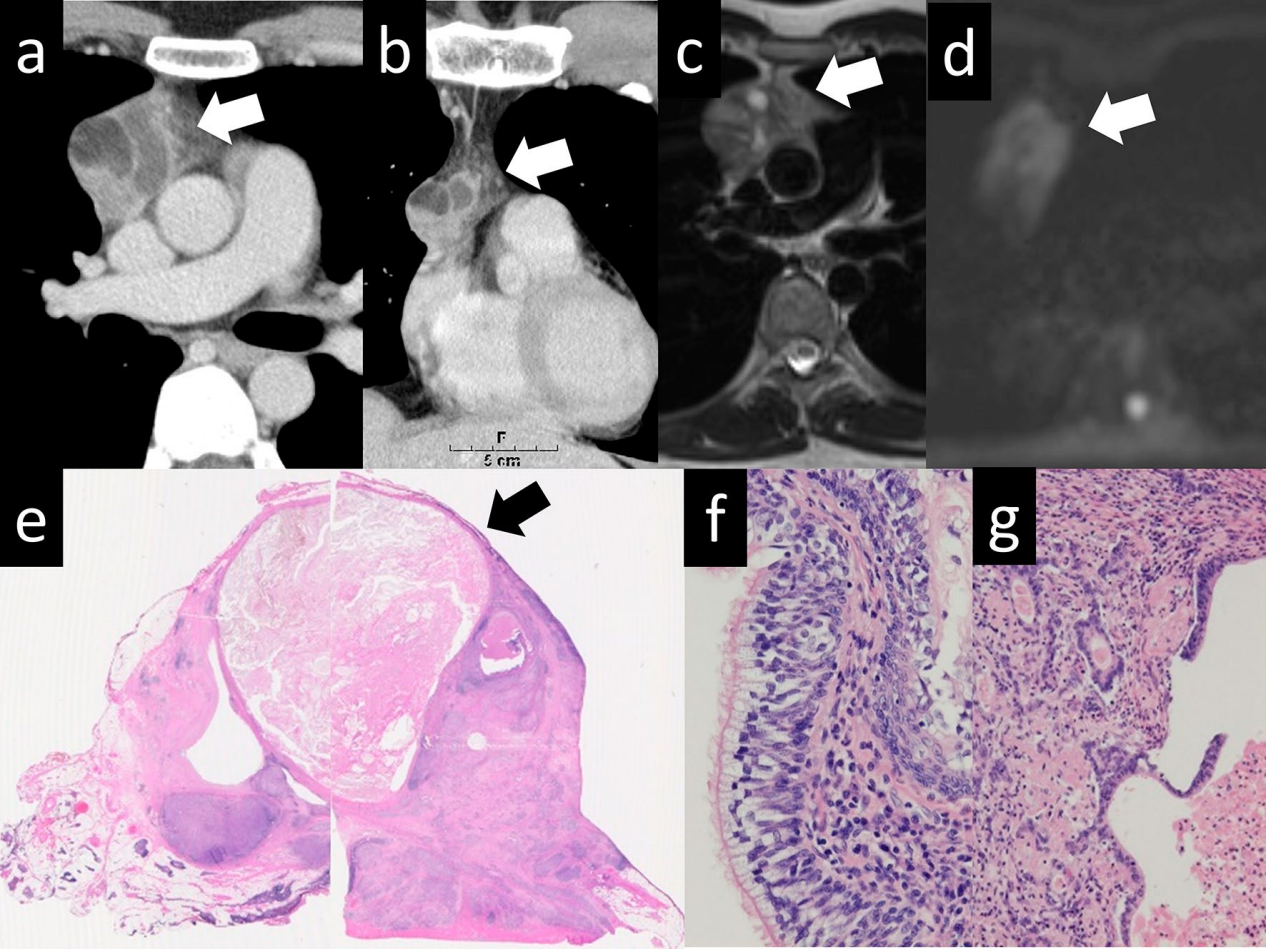
Germ cell tumor with somatic-type malignancy. A 32-year-old man with a germ cell tumor with somatic-type malignancy. **a**, **b** Chest CT shows a 2.5-cm nodule with solid, lipid, calcified, and cystic components (arrow). (**c**) The nodule shows heterogeneous intensity on T2WI (arrow). **d** The nodule shows slightly high intensity on DWI (arrow), and a low ADC value (1.2 × 10^–3^ mm^2^/s). **e**–**g** The pathological diagnosis was mature cystic teratoma with somatic malignancy (arrow). The pathological image **f** shows columnar pseudostratified epithelium suggestive of mature teratoma. The pathological image **g** shows the adenocarcinoma component, which has a round atypical nucleus and fibroglandular stroma (**e**: loupe image, **f**: HE staining, high-power image, **g**: HE staining, high-power image). CT, computed tomography; T2WI, T2-weighted imaging; DWI, diffusion-weighted imaging; ADC, apparent diffusion coefficient; HE, hematoxylin and eosin

Malignant GCTs account for 1–4% of all mediastinal tumors. GCTs with malignant components have been reported to occur in approximately 10% of cases (Fig. 17). Malignant nonseminomatous GCTs of the mediastinum often show elevated serum beta-human chorionic gonadotropin and/or alpha-fetoprotein levels. Similar to testicular nonseminomatous GCTs, more than 80% of mediastinal GCTs show amplification of 12p in the form of an isochromosome [33]. Mediastinal nonseminomatous GCTs have an adverse prognosis, similar to that of testicular nonseminomatous GCTs. In addition, mediastinal nonseminomatous GCTs are more prone to complications, such as the development of somatic-type malignancies, including adenocarcinoma, neuroendocrine tumors, malignant peripheral nerve sheath tumors, rhabdomyosarcomas, angiosarcomas, and hematologic neoplasms (acute leukemias, systemic mast cell disease), than testicular nonseminomatous GCTs [34]. Mediastinal GCTs typically manifest as large anterior mediastinal masses. On CT scans, they appear as heterogeneous masses with areas of necrosis, hemorrhage, and calcification. These tumors can extend into adjacent structures, compressing the surrounding mediastinal structures. MRI can provide valuable information about the extent and involvement of mediastinal GCTs. These tumors usually appear as large, heterogeneous masses on MRI, with variable signal intensities reflecting the presence of necrosis, hemorrhage, and calcification. MRI is helpful in assessing the invasion of adjacent mediastinal structures, including the heart, great vessels, and lungs. GCTs generally exhibit increased uptake of the radiotracer on PET scans. PET imaging is particularly useful in staging and detecting distant metastases.

### Lymphomas of the mediastinum

**Fig. 18:**
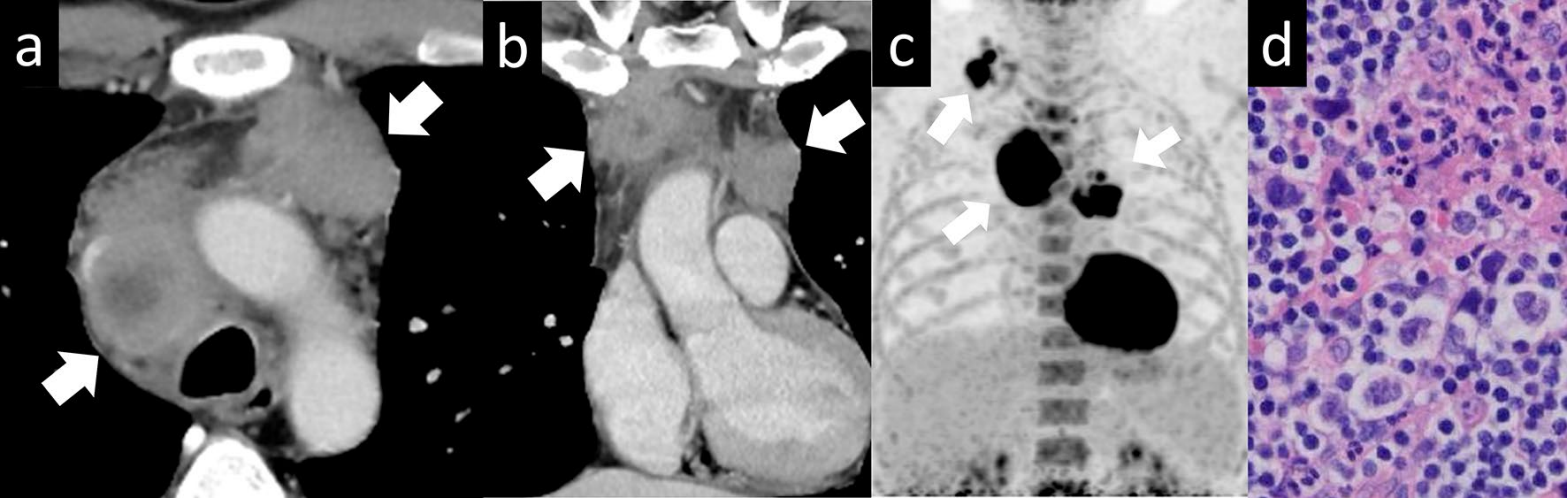
Hodgkin lymphoma. A 40-year-old man with Hodgkin lymphoma. **a**, **b** Contrast-enhanced-CT image shows masses with heterogeneous contrast enhancement in the mediastinal space (arrow). **c** Maximum intensity projection 18F-FDG PET images show a hypermetabolic conglomerate the mediastinal nodal mass on the right side of the aortic arch (SUVmax, 16.5) and mass on the left side (SUVmax 12.3) and additional foci of hypermetabolic lymphadenopathy disseminated throughout a right supraclavicular lymph node(SUVmax 9.8). **d** Biopsy with VATS was performed and nodular sclerosis classical Hodgkin lymphoma was diagnosed (HE staining, high-power image). The disease stage was Ann Arbor Stage IIA. CT, computed tomography; 18F-FDG PET, 18F-fluorodeoxyglucose positron emission tomography; SUVmax, maximum standardized uptake value; VATS, video-assisted thoracic surgery; HE, hematoxylin and eosin

**Fig. 19:**
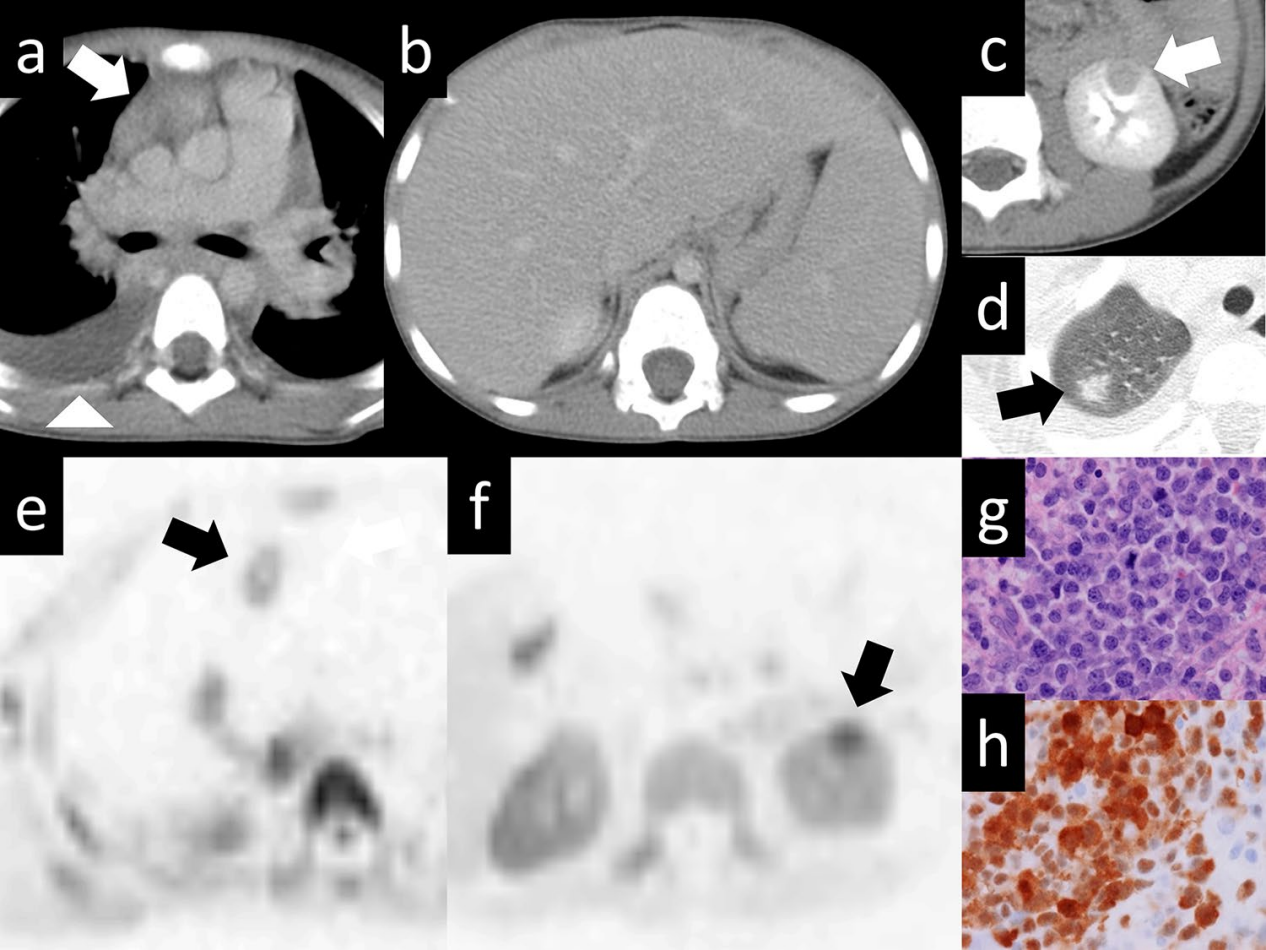
Non-Hodgkin lymphoma (ALK-positive anaplastic large cell lymphoma). A 5-year-old boy with ALK-positive anaplastic large-cell lymphoma (ALCL). **a**–**d** CT images show a mass in the anterior mediastinum (**a**), hepatosplenomegaly (**b**), a nodule in the left kidney (**c**), and a nodule in the right upper lobe (**d**) (arrow). **e**, **f** DWI (inverted black-and-white gray scale) indicates that the lesions exhibit reduced diffusion (arrow). **g**, **h** Predominant population of large cells with irregular nuclei (**g**). Malignant cells strongly expressed ALK (**h**). The diagnosis was mature T and NK-cell neoplasms (ALCL, ALK-positive) (**g**: HE staining, high-power image, **h**: ALK staining, high-power image). ALK, anaplastic lymphoma kinase; CT, computed tomography; DWI, diffusion-weighted imaging; HE, hematoxylin and eosin

Mediastinal lymphomas commonly present as part of a systemic disease or, less commonly, at the site of a primary disease. Lymphomas account for approximately 15% of all primary mediastinal masses and 45% of all anterior mediastinal masses in children [35]. Only 10% of lymphomas that involve the mediastinum are primary (mediastinal involvement is not part of the systemic disease), and most of them are classic Hodgkin lymphomas (CHLs) (approximately 60%) [36]. The mediastinum is commonly involved in the development of systemic lymphomas. Approximately 60% of all CHLs (Fig. 18) and 20% of non-Hodgkin lymphomas (Fig. 19) involve the mediastinum at the time of presentation [37]. Although patients present with systemic manifestations of lymphoma, most commonly constitutional symptoms, they do not often present with symptoms of mediastinal involvement [38]. Symptoms directly attributable to mediastinal involvement include retrosternal chest pain, superior vena cava compression with superior vena cava syndrome, dyspnea, and cough [39]. Mediastinal lymphoma usually originates in the thymus or lymph nodes, with a predilection for the anterior and middle mediastinum. The anterior mediastinal and paratracheal lymph nodes are the most frequently involved sites. Isolated hilar lymphadenopathy without mediastinal lymphadenopathy is rare. Involvement of the posterior mediastinum is also rare. The current WHO classification of neoplastic diseases of lymphoid tissues includes lymphomas and leukemias [40], which are categorized according to the cell of origin, with the main categories being B-cell neoplasms, T-cell and natural killer cell neoplasms, and Hodgkin lymphoma [41, 42]. Primary mediastinal lymphomas are most frequently observed in three histologic varieties, nodular sclerosing Hodgkin lymphoma, primary mediastinal large B-cell lymphoma, and lymphoblastic lymphoma [43]. Several distinctive types of lymphomas arise primarily in the thymus/anterior (prevascular) mediastinum, including nodular sclerosis classic Hodgkin lymphoma, primary mediastinal (thymic) large B-cell lymphoma, mediastinal grey zone lymphoma, T-lymphoblastic leukemia/lymphoma, and MALT lymphoma of the thymus, although rare [37]. Imaging modalities such as CT, MRI, and PET play an important role in the evaluation of primary mediastinal lymphomas. CT scans reveal a large anterior mediastinal mass with soft tissue density and irregular margins. Homogeneous enhancement after contrast administration, encasement of adjacent structures, and lymph node involvement are common findings. MRI typically shows a bulky anterior mediastinal mass with intermediate to hypointense signal intensity on T1-weighted images and heterogeneous hyperintensity on T2-weighted images. Contrast enhancement reflects vascularity and metabolic activity, while MRI helps evaluate invasion into adjacent structures. PET imaging demonstrates increased radiotracer uptake in the mediastinal mass, indicating active disease. It aids in staging, detecting distant metastases, and monitoring treatment response.

### Thymolipoma

**Fig. 20:**
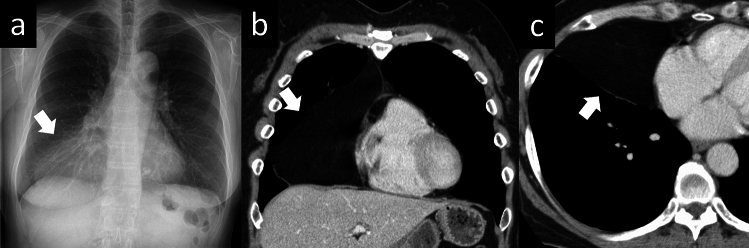
Thymolipoma. A 59-year-old male with thymolipoma. **a** Chest X-ray shows a unilateral ground-glass opacity in the right lower lung zone (arrow). **b**, **c** CT shows an adipose mass in the right hemithorax (arrow). CT, computed tomography

The mediastinum is an anatomical space that contains organs, major blood vessels, and a mixture of soft tissues. Most mediastinal tumors are associated with the thymus. Soft tissue tumors of the mediastinum are rare. Owing to their rarity, most cases of mesenchymal tumors of the mediastinum are only described in case reports or small case series [44, 45]. A thymolipoma is a very rare benign neoplasm of the thymus composed of mature adipose and thymic tissue (Fig. 20). It is a slow-growing mediastinal tumor that accounts for only 2–9% of all thymic tumors. Most patients with thymolipomas are asymptomatic and the tumors are usually detected incidentally, often during imaging assessment for respiratory tract infections. On CT, thymolipomas typically appear almost entirely fatty with some areas of inhomogeneous soft tissue density that represent thymic tissue. On chest X-ray, they appear as large anterior mediastinal masses. The larger tumors tend to hang down on one or either side of the pericardium, and being soft, they mold themselves to the adjacent mediastinum and diaphragm and often mimic cardiomegaly.

### Metastasis of the thyms

**Fig. 21:**
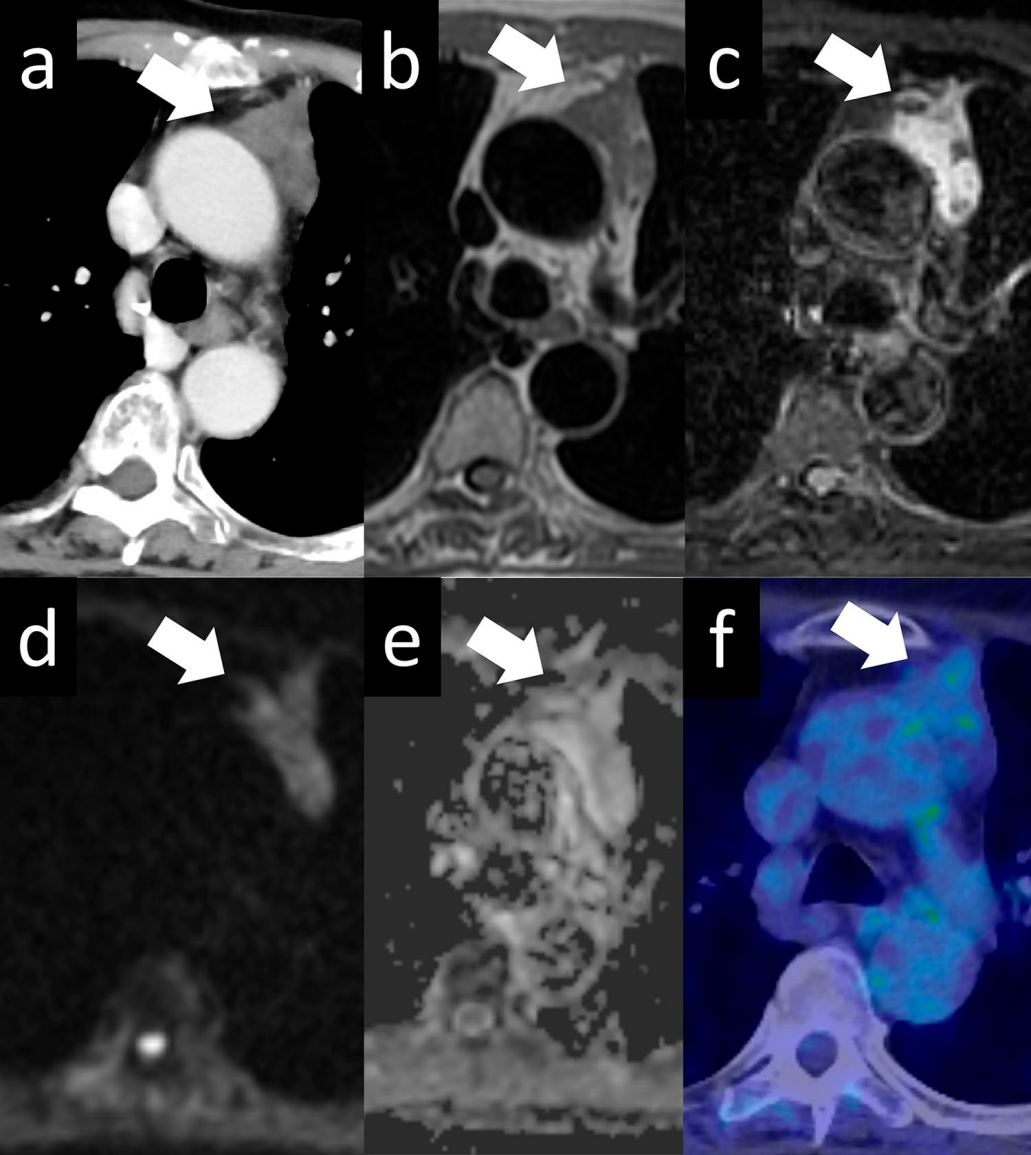
Thymic metastasis. A 70-year-old woman with a thymic metastasis from breast cancer. **a** CT shows a 3.7-cm irregular shaped mass (arrow). **b** The mass shows slightly high intensity on T1WI (arrow). **c** The mass shows high intensity on fat-suppressed T2WI (arrow). **d**, **e** The mass shows slightly high intensity on DWI (arrow). ADC value is 2.02 × 10^–3^ mm^2^/s. **f** 18F-FDG PET shows slightly FDG uptake in the perivascular mass (SUVmax = 2.5) (arrow). CT, computed tomography; T1WI, T1-weighted imaging; T2WI, T2-weighted imaging; DWI, diffusion-weighted imaging; ADC, apparent diffusion coefficient; 18F-FDG PET, 18F-fluorodeoxyglucose positron emission tomography; SUVmax, maximum standardized uptake value

Thymic metastases of malignant tumors are generally rare, and only a few cases with imaging findings have been reported [46]. Middleton et al. [47] reported 7 cases (7% of all cases) with thymic metastases in their review of 102 autopsy cases of malignancy. The thymic parenchyma contains a blood-thymus barrier that prevents the passage of antigens and malignant cells through the capillaries, resulting in a relatively low incidence of metastasis [48]. However, malignant cells can still spread into the thymus through the interlobular connective tissue, which contains vascular spaces, lymphatic vessels, and nerve tissue. The potential for misdiagnosing thymic metastasis exists due to the absence of typical radiological findings, which may occur depending on the degree of malignant cell infiltration within the thymus (Fig. 21). If there is a tendency toward enlargement and an irregular shape, it is possible that metastasis has occurred.

## Conclusion

This review focused on different imaging characteristics of the thymus gland, which changes due to a variety of reasons, ranging from aging to pathological conditions (Table 3). Owing to a complex combination of various mechanisms, differentiating between benign and neoplastic changes in the thymus is occasionally challenging. In addition, many patients with thymic tumors are asymptomatic. However, understanding the characteristic imaging findings for each condition or disease can lead to better understanding of the normal changes and diseases of the thymus. Radiologists need to understand that imaging plays a crucial role in the evaluation of thymic lesions because many thymic tumors are not easily diagnosed histopathologically.

**Table 3:** Summarizing the imaging findings of tumors in the prevascular compartment

Tumor type	Imaging findings
Thymic Cyst	Well-defined, thin-walled cystic lesion within the thymus Fluid-filled appearance with no solid components Smooth and thin cyst wall No enhancement on contrast-enhanced imaging
Thymoma	Well-defined anterior mediastinal mass Lobulated or irregular shape Heterogeneous enhancement on contrast-enhanced CT or MRI Presence of calcifications Invasion of adjacent structures (lungs, great vessels) in advanced cases
Thymic Carcinoma	Anterior mediastinal mass with irregular margins Heterogeneous enhancement on contrast-enhanced imaging Presence of necrosis or cystic areas Infiltration or invasion of adjacent structures
Thymic Neuroendocrine Neoplasm	Anterior mediastinal mass with variable morphology Heterogeneous enhancement on contrast-enhanced imaging Presence of solid and cystic components
Germ Cell Tumor	Anterior mediastinal mass with variable morphology Heterogeneous appearance with necrotic, cystic, or solid components Presence of fat or fluid–fluid levels in teratomas Enhancing solid components
Lymphoma	Bulky anterior mediastinal mass Homogeneous or heterogeneous appearance Diffuse or nodal involvement within the mediastinum Presence of mediastinal or other regional lymphadenopathy
Thymolipoma	Well-defined anterior mediastinal mass with fatty and soft tissue components Fat attenuation on CT imaging No invasion or compression of adjacent structures
Metastasis of the Thymus	Presence of multiple, irregularly shaped masses in the mediastinum Variable enhancement patterns depending on the primary cancer Adjacent lymphadenopathy or distant metastases may be seen
